# Damage Detection in FRP-Reinforced Concrete Elements

**DOI:** 10.3390/ma17051171

**Published:** 2024-03-02

**Authors:** Pranit Malla, Seyed Saman Khedmatgozar Dolati, Jesus D. Ortiz, Armin B. Mehrabi, Antonio Nanni, Jiayi Ding

**Affiliations:** 1 Department of Civil and Environmental Engineering, Florida International University, Miami, FL 33174, USA; skhed004@fiu.edu; 2 Department of Civil and Architectural Engineering, University of Miami, Coral Gables, FL 33146, USA; jdo72@miami.edu (J.D.O.); nanni@miami.edu (A.N.); 3 AtkinsRéalis, Miami, FL 33126, USA; jiayi.ding@atkinsrealis.com

**Keywords:** Fiber-Reinforced Polymer (FRP), ground-penetrating radar (GPR), ultrasonic testing (UT), phased array ultrasonic (PAU), non-destructive testing (NDT), FRP-reinforced concrete (FRP-RC)

## Abstract

Fiber-Reinforced Polymer (FRP) composites have emerged as a promising alternative to conventional steel reinforcements in concrete structures owing to their benefits of corrosion resistance, higher strength-to-weight ratio, reduced maintenance cost, extended service life, and superior durability. However, there has been limited research on non-destructive testing (NDT) methods applicable for identifying damage in FRP-reinforced concrete (FRP-RC) elements. This knowledge gap has often limited its application in the construction industry. Engineers and owners often lack confidence in utilizing this relatively new construction material due to the challenge of assessing its condition. Thus, the main objective of this study is to determine the applicability of two of the most common NDT methods: the Ground-Penetrating Radar (GPR) and Phased Array Ultrasonic (PAU) methods for the detection of damage in FRP-RC elements. Three slab specimens with variations in FRP type (glass-, carbon- and basalt-FRP, i.e., GFRP, CFRP, and BFRP, respectively), bar diameter, bar depths, and defect types were investigated to determine the limitations and detection capabilities of these two NDT methods. The results show that GPR could detect damage in GFRP bars and CFRP strands, but PAU was limited to damage detection in CFRP strands. The findings of this study show the applicability of conventional NDT methods to FRP-RC and at the same time identify the areas with a need for further research.

## 1. Introduction

The construction industry predominantly utilizes two structural materials: steel and concrete [1]. However, with the increasing demand for extended service life, reduced maintenance, enhanced resilience, and sustainability, the limitations of traditional construction materials (e.g., steel reinforced/prestressed concrete, structural steel, and timber) have become more evident. In response to these demands, Fiber-Reinforced Polymer (FRP) composites have emerged as a promising alternative, offering improved durability and performance and providing the potential for extended service life and reduced maintenance costs [2]. FRPs are composite materials composed of reinforcing fibers impregnated in a polymeric resin. The reinforcing fibers in the composite are the main load-carrying (reinforcing) elements, while the polymeric matrix or resin helps to form the desired geometry and transfers forces to and between the fibers. In general, the types of FRPs used in the construction industry based on the type of fibers are GFRP (glass-FRP), CFRP (carbon-FRP), BFRP (basalt-FRP), and AFRP (aramid-FRP) composites.

### 1.1. FRP-Reinforced Concrete (FRP-RC) Elements

Over the past three decades, FRP composites have gained significant popularity in civil engineering, attributed to mainly their increased durability, corrosion resistance, and higher strength-to-weight ratio [3]. They have been used as reinforcement for constructing new structures as well as rehabilitating existing ones. FRPs can be used either in conjunction with concrete elements or as stand-alone structural or non-structural elements in buildings as well as bridge structures. When used in conjunction with concrete elements, FRP application can be divided into two categories: (1) internal application with FRP bars/rods and strands/tendons for new FRP-reinforced/prestressed constructions and (2) external application with FRP laminates/plates/jackets, sheets/fabrics/wraps, and near-surface mounted (NSM) bars for the strengthening, retrofitting, and repair of existing structures. This paper focuses on the internal application of FRP composites, more specifically on the damage detection of FRP rods/strands embedded in concrete elements. For the sake of brevity, FRP bars/rods and strands/tendons are referred to as FRP bars in the following sections of this paper.

### 1.2. Advantages of FRP-RC Elements

Corrosion is one of the main issues that can compromise the serviceability and safety of conventional steel-reinforced/prestressed concrete structures. A 2002 Federal Highway Administration (FHWA) study conducted in partnership with the National Association of Corrosion Engineers (NACE) International, now known as the Association for Materials Protection and Performance (AMPP), estimated the average annual direct cost of corrosion for US highway bridges to be $8.29 billion [4]. A decade later, in 2013, NACE International estimated an increase in this cost to $13.6 billion per year [5]. Despite these estimates being decades old, the issue of corrosion persists, and it remains a primary cause of bridge deterioration in the US. The latest 2021 American Society of Civil Engineers (ASCE) infrastructure report card scored America’s bridges a low grade of C and emphasized the use of innovative materials such as ultra-high-performance concrete (UHPC), corrosion-resistant reinforcement, high-performance steel, composites, and improved coatings to increase the lifespan of the nation’s bridges [6].

FRP composites are one of such relatively new construction materials that are resistant to all the factors causing corrosion in steel-reinforced concrete (RC) structures, such as a decrease in concrete pH due to carbonation, chloride penetration, and the diffusion of halides and chemicals [7,8,9,10]. Further, FRP composites are not affected by electromagnetic disturbances from sources such as railroads with DC or AC traction, overhead power lines, and unbalanced currents from three-phase power systems, which contribute to the corrosion of metal structures and the deterioration of reinforced concrete [11]. Hence, the use of FRPs as reinforcement in concrete elements is strongly justified for locations where the corrosion of conventional steel reinforcement poses significant economic and safety risks [12]. 

Additionally, better mechanical performance, superior durability, and the environmental implications of the FRP composites [13,14] offer more flexibility for engineers to build structures that last longer. When compared to steel bars, FRP bars have significantly higher tensile strength [15], about one-fourth of the density of steel, and can achieve a longer service life [16]. Nevertheless, the application of FRP composites is associated with a higher initial cost, which is often quoted as one of the major drawbacks to its implementation. However, in recent years, the initial cost of GFRP bars has benefitted due to price fluctuation in the metal market worldwide since the mid-2020s and has even dropped due to the growth of the GFRP bar industry [17]. Further, despite the fact that FRP bars initially cost more than traditional steel bars, a life cycle cost study shows that they can rather be cost-effective in the long run [18]. Because of these factors, FRP bars are progressively becoming a reliable material in civil engineering. This is evident from a recent example of a coastal bridge fully reinforced with GFRP bars built in 2021 at the 23rd Avenue over Ibis Waterway located in Florida, USA, which is the second of its kind [3,19].

### 1.3. Damage in FRP-RC Elements

Although FRP bars offer improved durability and performance compared to steel in certain aspects, there are concerns about potential damage and defects in both FRP bars and FRP-reinforced concrete (FRP-RC) elements. Many of the serviceability issues related to conventional RC elements such as cracking, permeability, carbonation, chloride content, and concrete cover may not pose the same concern for FRP-RC elements. FRP bars and FRP-RC elements are prone to a unique set of defects as compared to their steel counterparts. For instance, the bond behavior of the FRP bar–concrete interface differs from that of the steel bar–concrete interface [20]. The bond failure of FRP bars not only occurs in the concrete but also inside the bars, unlike a steel bar [21,22]. Similarly, in a study conducted by Valentine [23], it was found that cracks are the predominant defect reported by the bridge inspectors in the inspection of FRP-reinforced bridge decks, which can be attributed to the low modulus of elasticity of the FRP bars. In this paper, the detectability of three different types of potential damage that might occur in the FRP reinforcements—rupture, debonding, and loss of cross-sectional properties—will be investigated. It should be noted that the term “potential damage” has been used due to the fact that, unlike steel bars, where corrosion is the obvious damage to be expected, there is very limited information on the damage that is possible in FRP bars, a relatively new, corrosion-resistant construction material. Additionally, this paper will also include the detection of damage in concrete such as delamination, cracks, and voids, which would be similar to traditional steel reinforced/prestressed concrete elements.

### 1.4. Inspection of FRP-RC Elements

The literature on the application of non-destructive testing (NDT) methods for the internal application of FRP is limited and scarce. There is no standard guide available for the inspection of FRP-RC elements [24,25,26]. This represents a knowledge gap that this research study attempts to address. Hence, although the use of FRP in highway infrastructures has been on the rise [27,28,29,30], the absence of reliable condition assessment methods for FRP-RC elements has significantly hindered its extensive application. Bridge engineers are hesitant to use materials that are difficult to detect and assess for maintenance. Therefore, there’s a pressing need for research into effective condition assessment techniques for FRP-RC elements, which could greatly encourage the adoption of FRP in future construction projects.

The inspection of FRP-RC elements is limited to detecting the initiation of FRP bars–concrete debonding [31,32] or the initiation of fractures in the FRP [33,34] rather than detecting the damage in the bars themselves. This is in most part because it was believed that FRP bars are undetectable or have low detectability, making it impossible to spot them effectively during an inspection. NDT techniques used for inspecting steel-reinforced concrete rely on identifying differences in specific properties, such as the dielectric constant and acoustic impedance, between steel and concrete. However, FRP reinforcements, unlike steel, exhibit properties similar to concrete that include non-conductivity and comparable density. These similarities introduce complexities in detecting/inspecting FRP, making it a more challenging task.

However, Ékes [35] demonstrated for the first time that ground-penetrating radar (GPR) can detect both CFRP and GFRP bars embedded in concrete and therefore concluded that it is a suitable tool for locating FRP bars on bridge decks. Another study conducted by the authors of this paper showed that the detectability of FRP bars/strands increased with the rise in the antenna center frequency of the GPR device and further showed that phased array ultrasonic (PAU) testing is also effective in detecting GFRP and CFRP strands [36]. PAU is sensitive in detecting air voids and hence it was effective only for FRP strands because of the air voids present within the twisted wires of strands and the uneven surface of the strands, unlike the smooth surfaces of bars. However, these studies do not give any information about the detectability of damage in FRP reinforcements using GPR and PAU. 

This paper explores the feasibility of employing commercially available GPR and PAU devices to identify damage in FRP bars embedded in concrete. These methods are selected among various NDTs because they are widely used in inspecting steel RC elements [24]. Further, this paper also aims to determine the detection of damage in the concrete elements reinforced with FRP using GPR and PAU devices. Three small-scale slabs were fabricated with damage simulated in bars and concrete to evaluate the feasibility of the chosen NDT method. The results of this study show that GPR devices can detect damage in FRP bars/strands and concrete. However, it was observed that PAU devices are effective only for detecting damage in CFRP strands along with steel bars and concrete. 

The results of this study can be utilized to drive further research on the non-destructive testing of FRP-RC elements and embedded FRP bars. One such prospective field of study in the future could be the use of NDT damage detection methods in conjunction with diagnostic load testing for bridges. Diagnostic load tests are performed to evaluate the integrity and performance of bridges and identify local damage areas based on the variations in measurements of deflections, strains, and vibration responses [19]. Once local damage areas are identified, NDT can be employed to perform a more thorough and refined damage assessment within those areas. When used together, NDT and diagnostic load testing can achieve efficient, comprehensive, and dependable damage detection and assessment of FRP bars embedded in concrete. These will provide owners with inspection options and help them in decision making regarding necessary countermeasures for ensuring the bridge’s safety and longer service life.

## 2. Experimental Program

### 2.1. Fabrication of FRP-RC Slab Specimens

To determine the capability of GPR and PAU in detecting defects in FRP reinforcements, three concrete slab specimens were fabricated and inspected. These slabs, measuring 36 inches in width, 36 inches in length, and 7 inches in depth, were fabricated with simulated defects in FRP bars and the concrete itself. The concrete mix design used for casting the slabs was determined following the specifications of the Florida Department of Transportation (FDOT) for “Class II 4500 Bridgedeck” concrete. The mixture included Type II cement with a water-to-cement ratio (w/cm) of 0.44, #57 stone as a coarse aggregate, and silica sand as a fine aggregate. Concrete cylinders were tested at 28 days following American Society for Testing and Materials (ASTM C39) standards [37,38] to verify the actual strength, resulting in an average compressive strength of 31.70 MPa with a standard deviation of 0.69 MPa (yielding a coefficient of variation of 2.2%).

The construction of slab specimens aimed to explore the effect of various factors in detectability, including the type of FRP bars/strands (GFRP, CFRP, BFRP), their diameters, the depths of embedment, and the type of defects. Table 1 shows the key characteristics of each slab specimen. Given the prevalent use of GFRP bars compared to other FRP bars in concrete reinforcement, one slab (Slab O) was reinforced only with GFRP bars at varying depths of embedment. According to American Concrete Institute (ACI) CODE-440.11-22 [39], the concrete cover for GFRP-reinforced members ranges between 0.75 inches and 3 inches, which guided the depth variations in the slab specimens to reflect the potential positioning of the top layer of FRP reinforcement. Slab P was designed to include two CFRP strands, a BFRP bar, and a steel bar (which serve as a benchmark for this investigation). This setup enables a direct comparison of the detectability of various FRP bars/strands against that of the steel bar under identical testing conditions. Additionally, the study extended to include Slab Q, which was designed to investigate the detection of simulated defects within the concrete itself.

**Figure 1:**
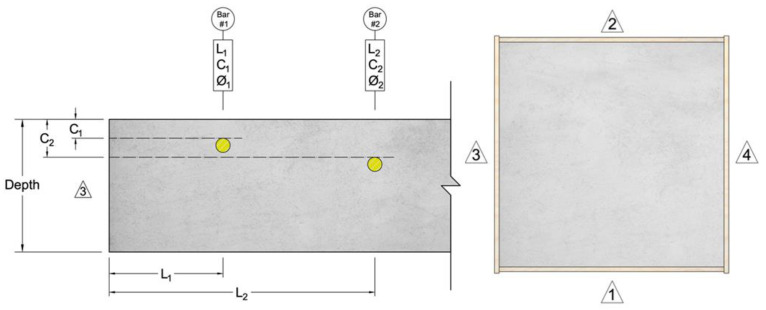
Labeling of slab specimens.

Each specimen was constructed and marked according to the layout depicted in Figure 1. Every slab is designated by an alphabet (O, P, and Q), and each side is assigned a number (ranging from 1 to 4). The direction of measurement is defined by the starting point and the endpoint numbers of the measurement.

### 2.2. Simulation of Defects in FRP Bars and Concrete

**Figure 2:**
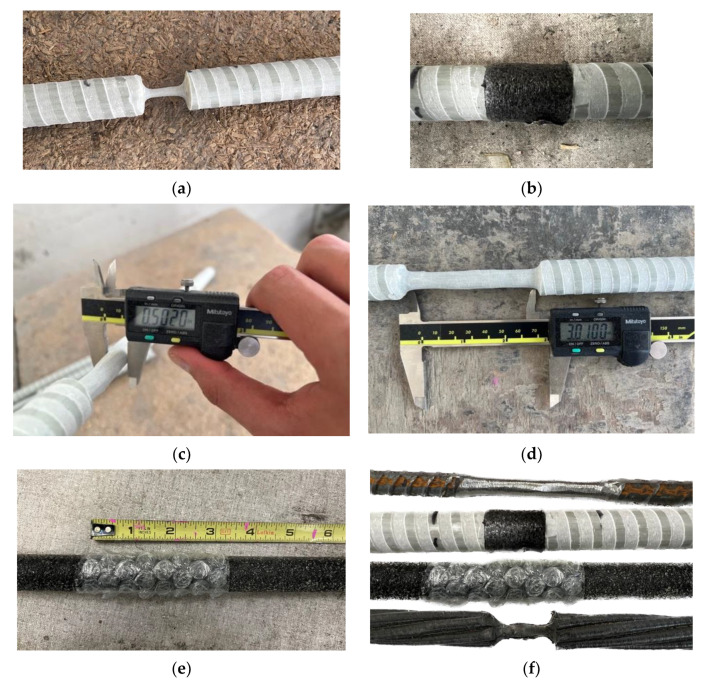
Damage introduced in bars. (**a**) Cross-section rupture, (**b**) covered rupture defect, (**c**) reduction in cross-section, (**d**) 3 inches of cross-section defect, (**e**) 3 inches of debonding defect, (**f**) different defects in bars.

**Figure 3:**
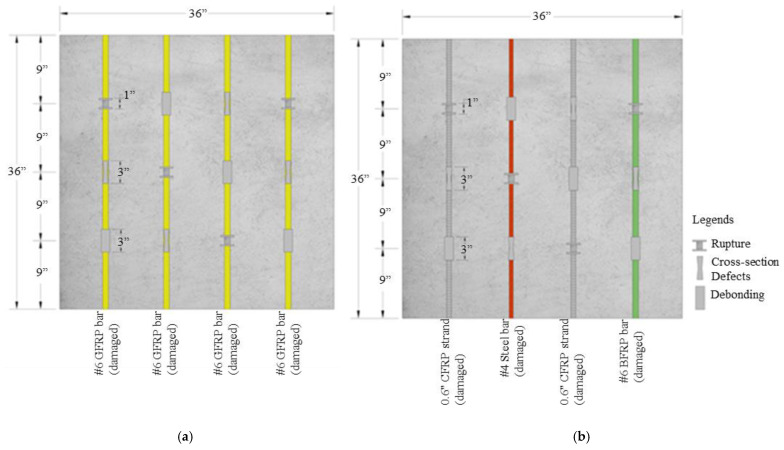
Slab specimen with simulated damage in bars, Slab O (**a**) and Slab P (**b**).

Slabs O and P contain defects in the bars. These defects correspond to bar rupture, a loss of cross-sectional properties, and debonding. To produce the bar rupture, the cross-section was reduced almost to breakage and then covered with a polyethylene tube to prevent the concrete from penetrating the defect and filling the void, as shown in Figure 2a,b. Cross-sectional property defects were produced by reducing the cross-section by 50% of its initial area and then covering it with polyethylene to prevent the concrete from filling the removed volume, as shown in Figure 2c,d. The loss of cross-sectional “properties” was simulated through the reduction in the cross-sectional “area” only for the experimental purpose of this paper. It should be noted that such an extreme reduction in the cross-sectional “area” of FRP bars may be unlikely, but the reduction in cross-sectional “properties” does occur under harsh environmental conditions. The aim is to explore the capability of NDT methods in detecting the variation in damage levels. Finally, the debonding defect was simulated by covering the bar with bubble wrap to generate a thin layer between the bar and the concrete (see Figure 2e,f). Figure 3 shows the location of the different defects and their dimension details.

**Figure 4:**
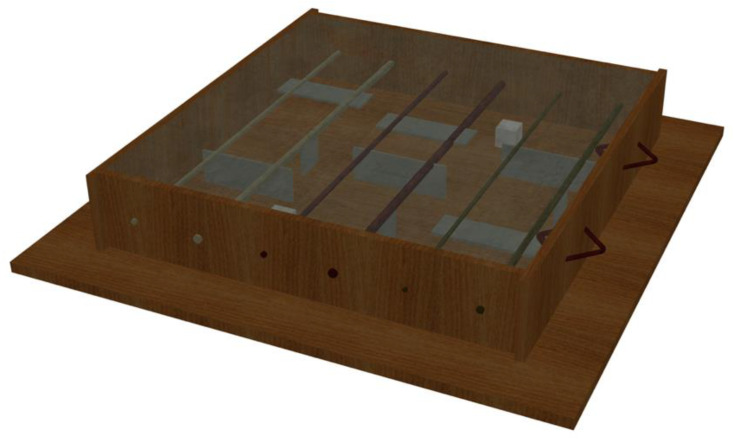
Three-dimensional scheme of Slab Q with simulated damage in the concrete.

**Figure 5:**
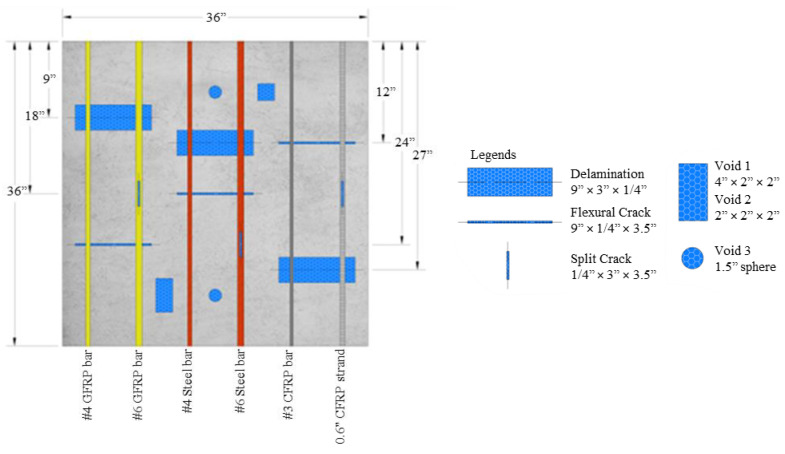
Dimension details of Slab Q with simulated damage in the concrete.

In Slab Q, four types of defects in the concrete were simulated to evaluate the feasibility of different NDT methods. Delamination, flexural and split cracks, as well as voids in the concrete were simulated in Slab Q using thin architectural polystyrene foam held in place with the use of epoxy, as shown in Figure 4 and Figure 5. This specimen includes steel, glass, and carbon FRP #4 and #6 bars.

Table 2 provides the geometrical details of the slab specimens, including the distance of the bars from the edge, cover depth, bar diameter, bar material, and slab thickness, following the conventions shown in Figure 1.

### 2.3. Non-Destructive Testing (NDT) Methods

**Figure 6:**
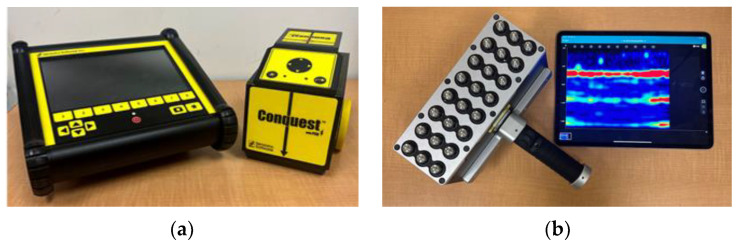
NDT devices used: (**a**) GPR device, (**b**) PAU device.

As mentioned in the introduction section, this paper aims to determine the detectability of damage in FRP reinforcements and FRP-RC elements using GPR and PAU devices (Figure 6). GPR is a real-time NDT method used to analyze the internal characteristics of civil structures. It operates on the principle that electromagnetic waves are reflected back when they hit a boundary between two materials with different dielectric constants [40,41]. The technique involves transmitting electromagnetic waves into the material under investigation and capturing the waves that are reflected from any irregularities within it. These irregularities could include boundaries between different materials, such as those between concrete and bars, or interfaces created by subsurface anomalies like voids, cracks, and instances of debonding or delamination in concrete [42]. Despite its potential, research on the application of GPR for inspecting FRP-RC elements is limited and its efficacy as a reliable NDT technique for this application remains unexplored. This study aims to investigate the feasibility of using GPR to detect damage in FRP bars.

The GPR system used in this experiment was a Conquest 100 Enhanced GPR (Sensors and Software Inc., Mississauga, ON, Canada) with a monostatic GPR antenna with a center frequency of 1000 MHz. GPR tests were carried out using both individual line scans and comprehensive grid scans. The line scans served as an initial survey to provide a preliminary understanding of the internal structure, including the orientation of reinforcements and the depth of exploration, by producing a cross-sectional image along the scan direction. However, this method proved to be time-consuming and labor-intensive due to the need to interpret multiple line scans. To streamline the process, grid scans were introduced, involving systematic GPR data collection along a predefined grid covering the test area. The grid’s line spacing, set at 2 inches for this study, directly influenced the resolution of the collected data, with closer spacing yielding higher-resolution images and easier data interpretation. The data from grid scans produced depth slice images, offering a cross-sectional view parallel to the specimen’s surface, facilitating a detailed analysis of the internal features.

PAU is the other NDT method being investigated in this study which is simply an advancement over the conventional ultrasonic testing (UT) method. Similar to GPR, UT is based on the principle that the ultrasonic waves are reflected back upon encountering a boundary between two materials with different acoustic impedances. These ultrasonic waves are generated and received by transducers that convert electrical or optical signals into ultrasonic waves and vice versa. A PAU setup is achieved by arranging multiple transducers in an array (Figure 6) and activating them sequentially with slight delays, allowing the individual waves to interfere constructively and destructively [43]. This arrangement enables the focusing and steering of the ultrasonic waves. PAU offers test results that are easy to interpret, a scanning rate of 5 to 10 times, and better resolution, reliability, portability, and mobility than conventional UT [44,45]. However, one of its major drawbacks is the uncertainty associated with its application as it has not yet been fully tested for inspection of FRP-RC elements.

The PAU device used for this experiment was a Pundit Live Array Pro device (Screening Eagle Technologies, Zurich, Switzerland) with 8 × 3 dry-contact Pundit array transducers. The PAU line scan was performed by moving the array of ultrasonic transducers along a specified line of inspection. Each scan at distinct positions was combined to form a continuous cross-sectional image perpendicular to the surface. For area scans, a stripe scan technique was employed, moving the transducers perpendicular to the line of inspection. Each stripe scan produced a line scan with a width equal to that of the PAU device, and these scans were stitched together to create a depth slice view. This view represents a cross-section parallel to the scanned surface, which can be further developed into a comprehensive 3D iso-surface model. A previous study by the authors [36] can be further explored for in-depth information on the applicability of GPR and PAU techniques for the inspection of structural elements reinforced with FRP.

## 3. Results

### 3.1. Ground-Penetrating Radar (GPR)

**Figure 7:**
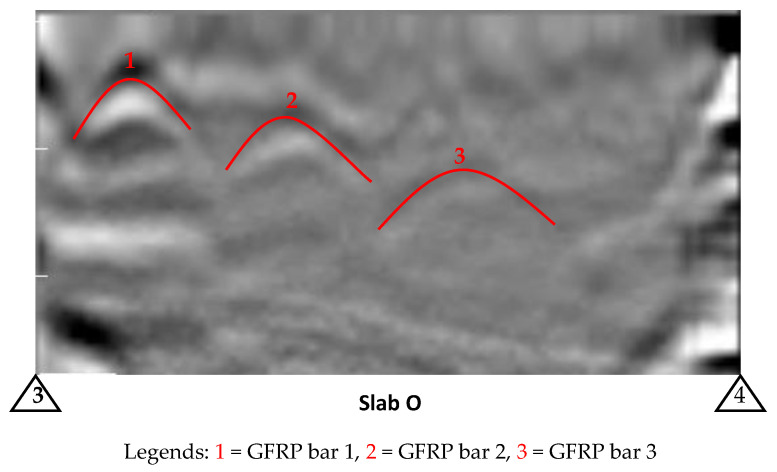
Line view for bar detection carried out before defect/damage detection (Red markings are superimposed/added on test results to indicate distinctive features).

In order to detect damage in FRP bars, it is first important to determine whether the GPR device is able to detect the bars itself. The line scans (B-scans) of the slabs O, P, and Q were obtained for the sole purpose of checking bar detectability before taking the detailed area scan which can be used in the field inspection for the real-time detection of damage. Figure 7 shows the GPR response of a line scan collected perpendicular to the embedded bars in the longitudinal direction (from the reference edge 3 to 4) over Slab O. The top of the hyperbolic shape (i.e., inverted U shape) in the figure indicates the location of the bars.

**Figure A1:**
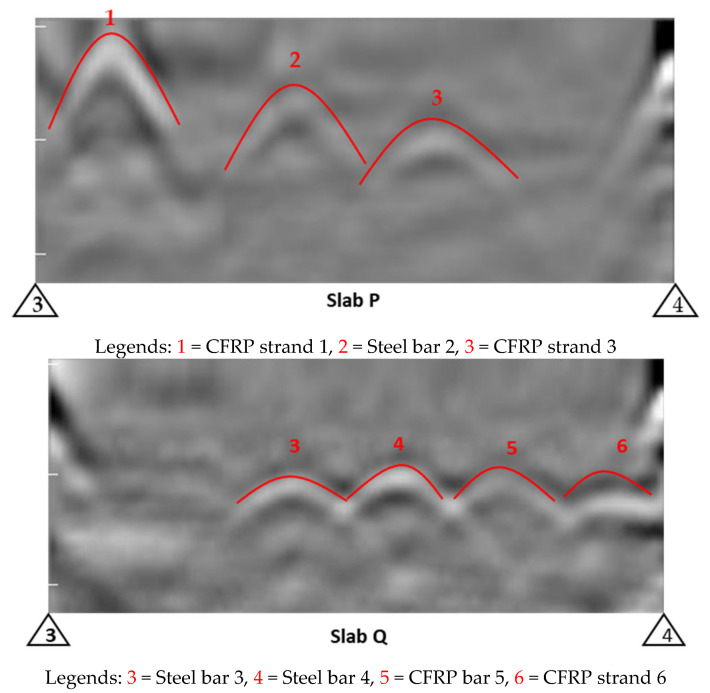
Line views for bar detection carried out before damage detection (for Slabs P and Q).

The GPR line scan of only one of the slabs (Slab O) is presented in this paper for the sake of brevity and Table 3 summarizes the results of the line scan test of all the other slabs. The line scans of Slab P and Q are presented in Appendix A, Figure A1. 

**Figure 8:**
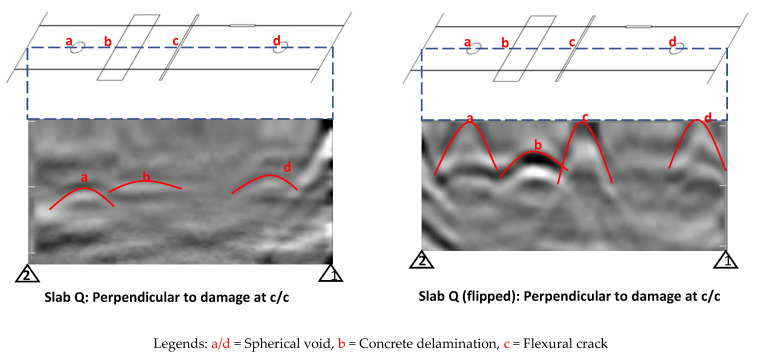
Line views for concrete defects/damage at c/c of Slab Q (red markings are superimposed/added to test results to indicate distinctive features).

It was observed that GPR could not detect the #6 GFRP bar in Slab O (Bar 4) and the #6 BFRP bar in Slab P (Bar 4), which have a concrete cover of more than 3.5 inches. Hence, it is clear that GPR would not be able to detect bar damage with a larger concrete cover. Slab Q, on the other hand, did not have any damage in the embedded bars but the damage was introduced to the concrete in the vicinity of the bars (in some cases below those bars). In addition to taking line scans perpendicular to the embedded bars in Slab Q, line scans were also taken at the center of the slab parallel to the bars to detect the damage introduced to the concrete. Two such line scans were taken, one from the top surface and the other from the bottom surface (after flipping the slab upside down), which are shown in Figure 8. It can be seen that more damage was visible from the bottom surface than from the top surface because the damage was closer to the bottom. The line scans conducted from the top surface could not detect vertical damage (flexural and split cracks) at all. Moreover, it can be seen that the hyperbolas for vertical damage (damage “c” in Figure 8) are narrower and taller than those for horizontal damage (damage “b” in Figure 8), which are wider and shorter. The damage “a” and “d” in the line scan carried out from the bottom surface (flipped Slab Q) appears to have been located at the surface, which is erroneous. This could be attributed to the use of small polystyrene foam cubes as a base for securing this damage (ping pong balls representing voids) at the bottom of the formwork.

**Figure 9:**
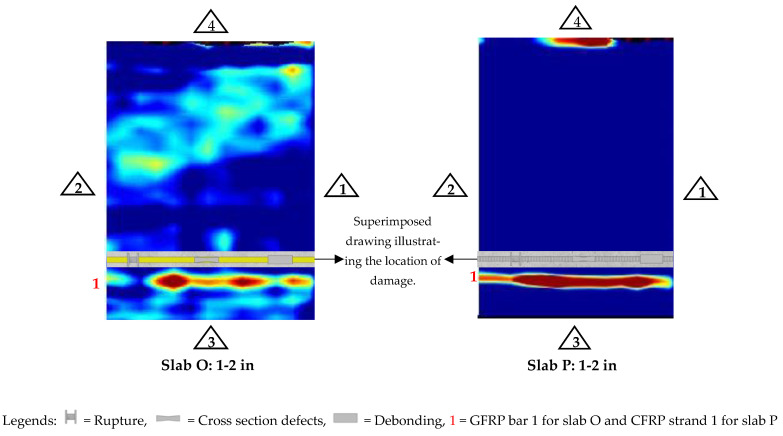
Depth slices of slabs for damage detection in bars.

**Figure 10:**
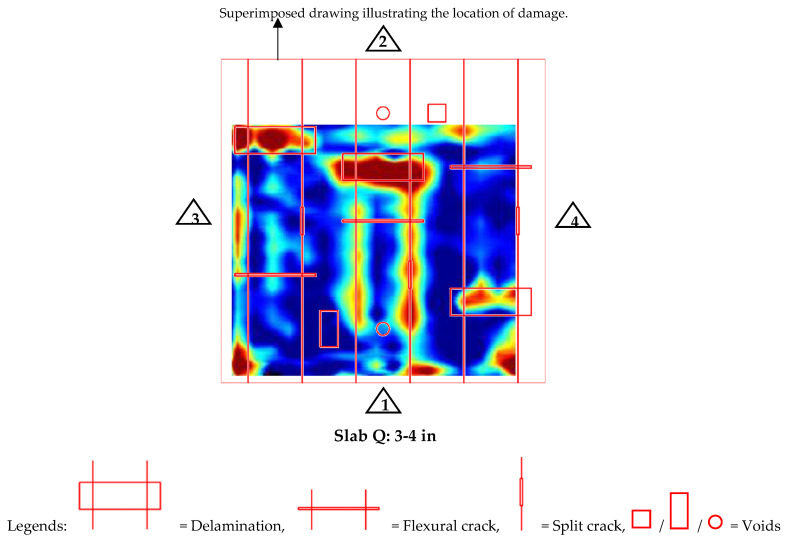
Depth slices of slabs for defect/damage detection in concrete.

Next, depth slices (C-scans) taken with GPR to determine the damage detectability for each of the constructed slabs (Slab O, P, Q) are shown in Figure 9 and Figure 10. To generate the depth slices, data collection was conducted longitudinally from reference edges 3 to 4, with a consistent spacing of 2 inches.

**Figure A2:**
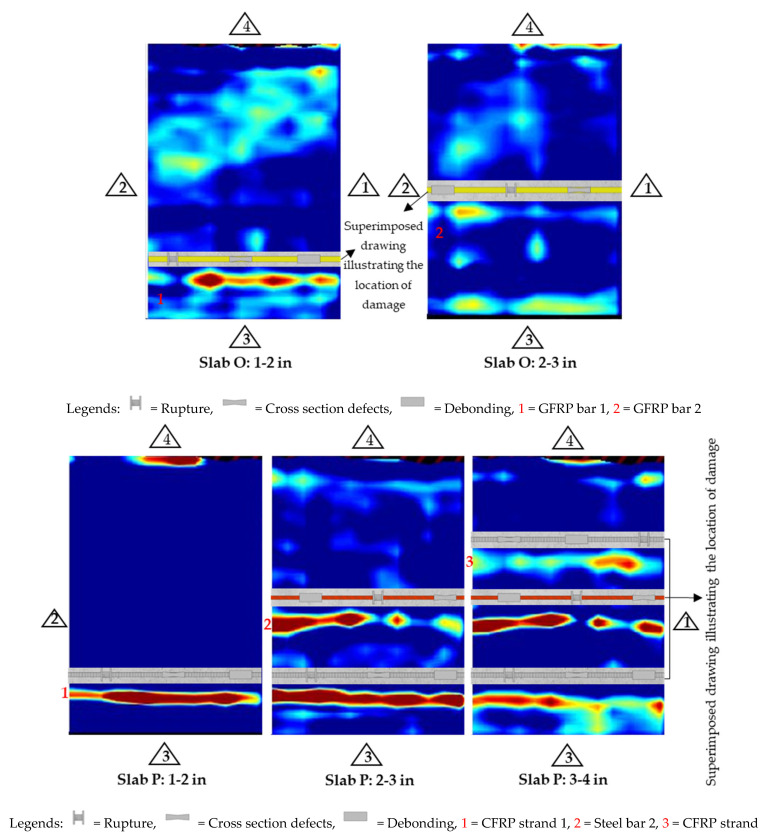
Depth slices of slabs O and P for damage detection in all the bars.

In Slab O with damaged GFRP bars, GPR successfully identified various forms of damage, including rupture, cross-sectional properties reduction, and debonding in Bar 1, as shown in Figure 9. However, the damage in Bar 2 is not quite distinguishable (see Appendix A, Figure A2). The bar itself is not as clearly visible as Bar 1, which is also because the already weak signal from GFRP bars (compared to steel bars) becomes even weaker with increasing depth. Further, while Bar 3 was detectable during the line scan, it was not visible in the depth slice due to its comparatively weaker signals relative to those from Bars 1 and 2. Thus, it was observed that the GPR exhibited limitations in detecting GFRP bars situated at greater depths, rendering it ineffective in identifying damage in these deeper-embedded GFRP bars. Consequently, it can be concluded that GPR’s damage detection capability is primarily confined to GFRP bars located at shallower depths.

The GPR depth slice results for Slab P varied in efficacy for detecting damage across different reinforcement materials. Specifically, for the CFRP strand located near the surface, GPR failed to identify any damage (see Figure 9). However, for the CFRP strand positioned at a depth of 3.5 inches, there were faded indications of damage (see Appendix A, Figure A2). In contrast, GPR exhibited comprehensive damage detection capabilities for the steel bar, identifying all present damage (see Appendix A, Figure A2). The BFRP bar presented a unique challenge; GPR was unable to detect it, particularly in the presence of dominant signals from the steel bar and CFRP strands. Consequently, damage in the BFRP bar remained undetected. However, it is worth noting that if the slab was only reinforced with BFRP bars, it is possible that GPR might have been able to detect damage in BFRP bars to a certain degree.

**Figure A3:**
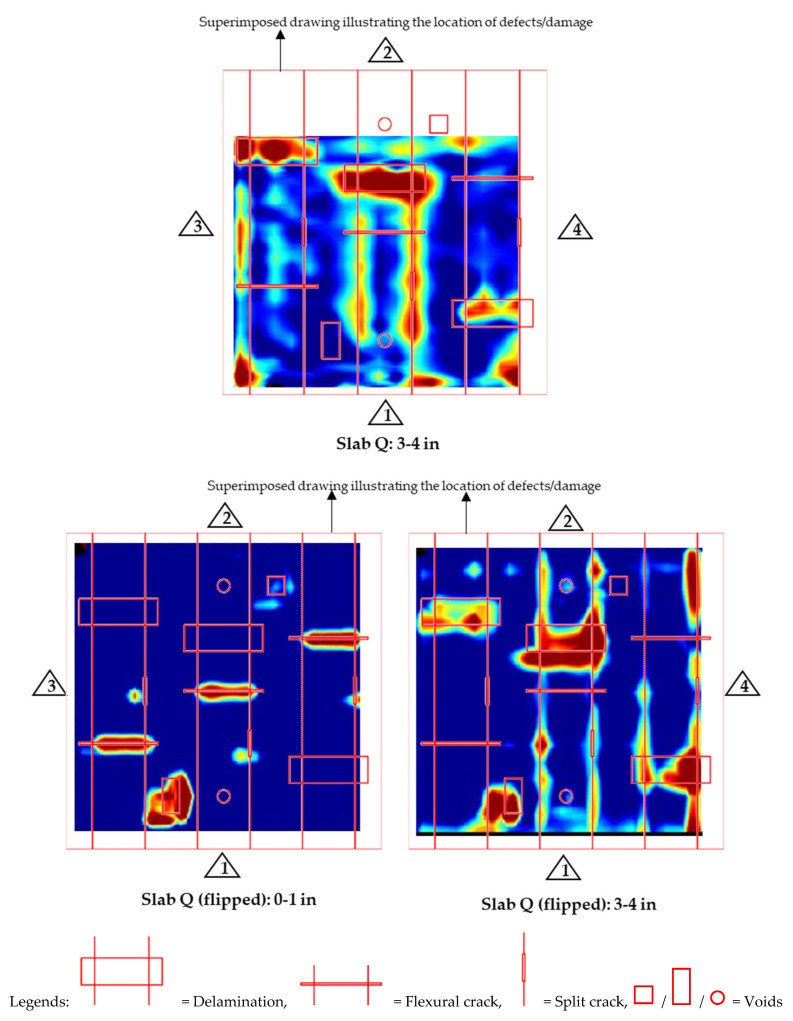
Depth slices of slab Q for damage detection in concrete (from top and bottom surface).

The depth slice of slab Q shown in Figure 10 illustrates GPR’s capability to clearly identify delamination within the concrete slab. However, its performance was limited when it came to the detection of deeper vertical cracks. In contrast, when the GPR test was conducted over the bottom surface of the slab, it proved adept at identifying shallower vertical cracks and voids (see Appendix A, Figure A3). These findings underscore the nuanced performance of GPR in detecting different types of defects in concrete, highlighting its selective sensitivity to various forms of damage depending on their nature and depth within the elements.

### 3.2. Phased Array Ultrasonic (PAU)

**Figure 11:**
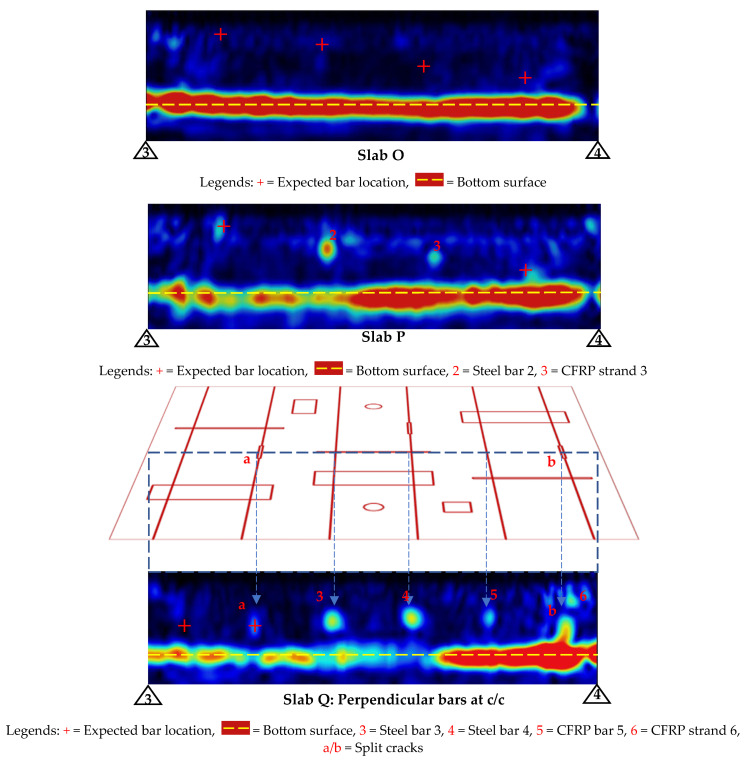
PAU line views for bar detection before damage detection.

The PAU line scans were taken with a similar approach used for GPR line scans for first detecting the bars as shown in Figure 11. For Slab O, no GFRP bars were detectable, and for Slab P, the steel bar (Bar 2) and the CFRP strand (Bar 3) were clearly detectable. Similarly, for Slab Q, the steel bars (Bars 3 and 4) were clearly detectable. Further, there were also some indications of vertical cracks (labeled a and b in Figure 11).

**Figure 12:**
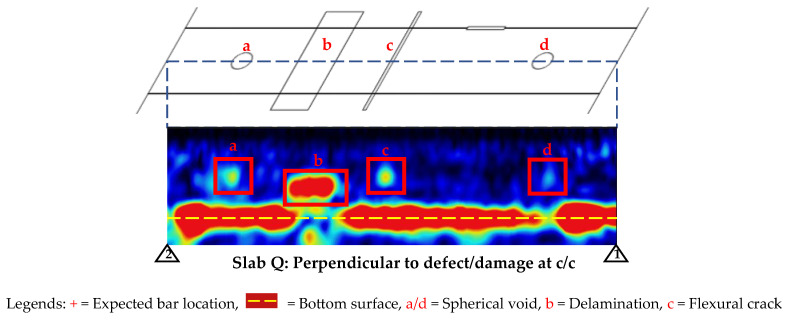
Line view for concrete defects/damage at c/c of Slab Q using PAU.

In addition to performing line scans perpendicular to the embedded bars in Slab Q, line scans were also conducted at the center of the slab parallel to the bars to detect the damage introduced in the concrete, as shown in Figure 12. It can be seen that the horizontal delamination (labeled as b in the figure) was distinctly visible but the vertical cracks (labeled as c in the figure) were only visible as a dot at the top point of the crack where it initiates (instead of being visible as a vertical line). Similarly, the voids (labeled as a and d in the figure) were also visible but not as distinct as the delamination, which could be because they were smaller in size.

**Figure 13:**
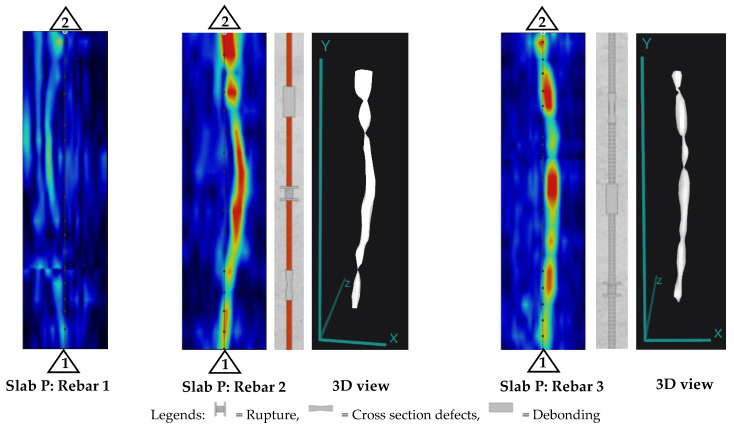
Depth slices of slabs for damage detection in bars using PAU.

**Figure 14:**
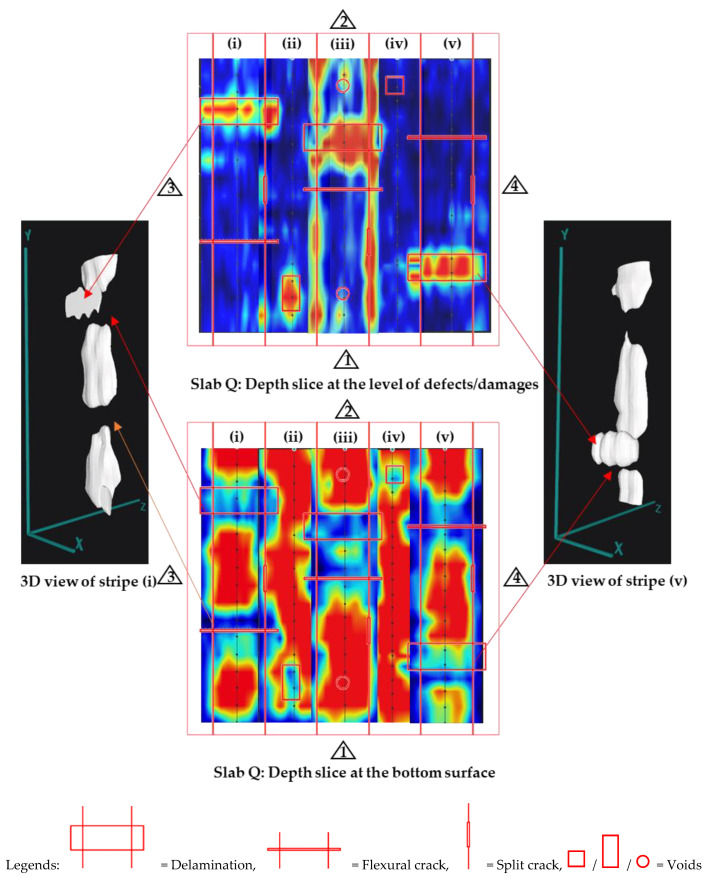
Depth slices of slabs for damage in concrete detectability tests using PAU.

As expected, since GFRP bars could not be detected using PAU (as mentioned earlier in the PAU line scan results), the area scans over Slab O did not yield any damage detection. However, the depth slices and 3D view of PAU tests for Slabs P and Q (shown in Figure 13 and Figure 14) produced some promising results.

It can be observed that Bar 2 and Bar 3 in Slab P do not appear to be straight. This is because Bar 2 (steel bar) had a kink because of a rupture at its mid-length resulting in a shift during casting, making it look like a bent bar. Similarly, in the case of Bar 3 (CFRP strand), the detected shape appears to be curved since these strands were flexible and curved outwards and downwards (sag) during the concrete pouring process. The CFRP strand had the tendency to bend to its transported coiled shape. It should also be noted that although it seemed the CFRP strand closer to the top surface (Bar 1) was visible in the line view (Figure 11), upon conducting the area scan over the strand, the detection proved not distinct enough for the entire strand (Figure 13). Only a portion of the strand at the mid-span was visible, which could be due to the fact that it sagged at the center with the weight of the concrete during casting, therefore increasing its depth from the top surface and making it free of surface reflections. These nuances of the PAU test results obtained from Slab P further illustrate the precision of PAU devices in detecting the geometrical orientation of the steel bar and CFRP strands embedded in concrete.

Similarly for Slab Q, while the CFRP bar (Bar 5) and CFRP strand (Bar 6) seem to be visible in the line scan (Figure 11), they were not visible on the depth slices (Figure 14), which could be because the signals from the damage in concrete (delamination) dominated over the weaker signal from the CFRP bar/strand.

In the context of damage detection in FRP bars, from the results of Slab O, it is evident that the PAU test cannot detect embedded GFRP bars at any depth and hence it also cannot detect any damage in GFRP bars. In slab P, the PAU was unable to detect the CFRP strand situated closer to the top surface. Additionally, its capability was limited when it came to the BFRP bar, rendering it ineffective in identifying damage within these BFRP bars. In contrast, PAU displayed comprehensive detection capabilities for the steel bar, with all damage, including the rupture at the bent position, being clearly identifiable. This clarity in damage detection was further enhanced when viewed in a 3D perspective. However, the damage in the CFRP strand at an intermediate depth, specifically Bar 3, was not as readily discernible as those in the steel bar. The presence of damage in this strand could be interpreted from the non-uniform color scale along the detection path, with red being the stronger signal and green to yellow being the weaker signal associated with defective/damaged areas.

Finally, for Slab Q, the PAU was adept at clearly identifying horizontal delamination and voids within the slab. Regarding the detection of vertical cracks in concrete (flexural and split cracks), although the direct detection proved challenging, these cracks could be inferred from the observed discontinuities or gaps present at the slab’s bottom reflection [46], shown at the bottom center image and the 3D views shown in Figure 14. The bottom surface of the slab specimen is not visible below delamination and cracks because these discontinuities prevent the propagation of ultrasonic waves below them and may even trap the waves to bounce back and forth between them and the top surface, resulting in multiple equally spaced reflections [47].

## 4. Discussion

The test results are summarized in a comprehensive test result matrix shown in Table 4. From the table, it can be seen that except for the case of the damaged BFRP bar, there is no other case where both GPR and PAU collectively have the label “ND” (not detectable). Therefore, the application of these two NDT methods together will not miss the detection of damage in internal FRP bars/strands and concrete. Hence, all the test parameters are found to be either detectable, “D”, or to have limited detectability, “LD” (limitation based on depth), by at least one of the GPR or PAU devices. In other words, in case a parameter is not detectable by GPR, then the detectability or limited detectability can still be ensured using PAU and vice versa. Thus, using both the GPR and PAU would be the best option to inspect FRP-RC elements. Otherwise, detectability can still be achieved using just one of these devices for the parameters mentioned as “LD” and “D” in Table 4.

However, there are some limitations related to the research conducted in this paper that warrant further investigation and consideration in future research studies concerning nondestructive testing techniques for the inspection of FRP-RC elements. It should be noted that the results shown in Table 4 have been obtained under laboratory conditions and the range of parameters used for the test specimens and test methods may not be applicable generally. For example, certain techniques were used to simulate the damage in FRP, e.g., the reduction in cross-section by grinding out the material and taping the bar to simulate debonding. Nevertheless, because the specimen and test conditions aimed to be as practical as possible, the results can provide credible guides for the use of NDT methods for FRP-RC elements. Regarding the inability to detect damage in the BFRP bar in Slab P, further experiments need to be conducted on slab specimens only reinforced with BFRP bars (similar to Slab O) before coming to a conclusion about the detectability of damage in BFRP bars. The low frequency 1 GHz GPR device used in this research was not able to detect the BFRP bar but a previous study by the authors [36] has proved that it can be detected using higher frequency GPR devices. Future research on detecting damage in BFRP bars should focus on using high-frequency GPR devices to detect several types of simulated damage with variations in parameters such as depth, extent, and type of damage. Since BFRP bars have gained popularity in recent years, it is justifiable to dedicate future research to the detectability of damage in BFRP bars to gain confidence among engineers in its use as a reinforcing material. Additionally, the scope of this study was limited to the real-time test results obtained from the proprietary software that comes with the commercially available GPR and PAU devices used in this study. This method of data collection represents the actual field conditions faced by the inspectors, who do not have access to complicated post-processing software at the inspection site location. Future research on refining these NDT techniques could even explore the integration of artificial intelligence and machine learning [48,49] for improved data analysis on remotely sensed data. Similarly, this study is limited to only three specimens with limited variation in depth of bars up to 4.5 inches, which can be overcome in future studies by conducting experimental verification on several specimens with a wider range of test parameters to collect more data for a statistically sound validation. One such extension could be testing the ability of the NDT devices to detect damage in multiple layers of reinforcements, as this study is limited only to the investigation of the first layer of reinforcement at the selected cover depth.

## 5. Conclusions

This study investigated the application of GPR and PAU in detecting damage in the FRP-RC elements. Three slab specimens with variations in several parameters, such as FRP type (GFRP, CFRP, BFRP), bar diameter, bar depth, and defect types were fabricated to determine the limitations and detection capabilities of these two NDT devices. Damage in the FRPs was simulated by a reduction in the cross-section to represent changes in cross-sectional properties and by wrapping the bars with tape to represent debonding. Foam pieces, flat and solid, were used to simulate the damage expected in the concrete. The findings of this study generally conclude that the combined use of GPR and PAU can detect potential internal defects associated with FRP-RC elements, as well as delamination, cracks, and voids in concrete.

The findings of this study have contributed significantly to the field of non-destructive testing (NDT) for FRP-RC elements. The successful demonstration of the combined use of GPR and PAU methods in detecting a variety of damage in FRP-RC elements under laboratory conditions lays the groundwork for future research and practical applications. The practical implications of these findings could involve the development of guidelines for the application of GPR and PAU methods in the inspection of FRP-RC elements in real-world scenarios, potentially improving the safety, maintenance, and durability of such structures. The specific conclusions of this paper include:

- Damage associated with the FRP-RC elements can be categorized as damage in the FRP reinforcements and those in the concrete. The potential types of damage in the FRP bars were identified as ruptures, loss of cross-sectional properties, and debonding, while the internal damage in concrete includes delamination, cracks, and voids.
- GPR could detect damage in GFRP bars, CFRP strands, steel bars, and all the internal damage introduced in concrete. It was not able to detect damage in BFRP bars in the experimental setup considered in this study, but there is a possibility that a higher frequency GPR device may be able to detect damage in BFRP bars, which is to be investigated in future studies.
- PAU showed limitations in its capability to detect damage in GFRP and BFRP bars but performed well in detecting damage in CFRP strands, steel bars, and concrete.
- Using GPR and PAU testing together would be the best option to inspect FRP-RC elements as the damage missed by one method would be detectable by the other. However, detectability (except for BFRP bars) can still be achieved using just one of these devices but with some limitations on the depth of the FRP.
- There may be some limitations related to the research conducted in this paper that may warrant further investigation. The experiments were under laboratory conditions and for a range of parameters for specimens and test methods and may not be applicable generally. Nevertheless, because the specimen and test conditions were chosen to be as practical as possible, e.g., FRP type, sizes, and concrete cover similar to actual values, the results can provide a credible guide for the use of NDT methods for FRP-RC elements.

## Figures and Tables

**Table 1:** Identification of small-scale concrete slab specimens.

Slab Specimen	Slab ID	Bar Diameter	No. of Bars
Slab with damaged GFRP bars	Slab O	#6 GFRP bars	4
Slab with damaged CFRP strands, BFRP bar, and steel bar	Slab P	0.6″ CFRP strands, #4 steel bar, and #6 BFRP bar	4
Slab with damage in concrete	Slab Q	#4 and #6 GFRP bars, #4 and #6 steel bars, #3 CFRP bar, and 0.6″ CFRP strand	6

**Table 2:** Reinforcement/dimension details of slab specimens.

Slab ID	Parameter (Symbol/Units)	Reference Side	Bar 1	Bar 2	Bar 3	Bar 4	Bar 5	Bar 6
O	Distance to edge reference (L/inch)	3	6.0	14.0	22.0	30.0		
	Cover depth (C/inch)		1.0	2.0	3.5	4.5		
	Bar diameter (ϕ/inch)		#6					
	Bar material (T)		Glass					
	Slab thickness (h/inch)		7.0					
P	Distance to edge reference (L/inch)	3	6.0	14.0	22.0	30.0		
	Cover depth (C/inch)		1.0	2.0	3.5	4.5		
	Bar diameter (ϕ/inch)		0.6”	#4	0.6”	#6		
	Bar material (T)		C-Std *	Steel	C-Std *	Basalt		
	Slab thickness (h/inch)		7.0					
Q	Distance to edge reference (L/inch)	3	3.0	9.0	15	21	27	33
	Cover depth (C/inch)		3.3	3.1	3.3	3.1	3.3	3.2
	Bar diameter (ϕ/inch)		#4	#6	#4	#6	#3	0.6’
	Bar material (T)		Glass	Glass	Steel	Steel	Carbon	C-Std *
	Slab thickness (h/inch)		7.0					

* C-Std. (i.e., CFRP strands) labeled as Bars 1 and 3 in Slab P and Bar 6 in Slab Q.

**Table 3:** Summary of bar detectability for damage detection.

Slab ID	Bar 1	Bar 2	Bar 3	Bar 4	Bar 5	Bar 6
O	✓	✓	✓	X	-	-
P	✓	✓	✓	X	-	-
Q	X	X	✓	✓	✓	✓

Note: ✓ = detectable, X = not detectable.

**Table 4:** Effectiveness of GPR and PAU methods for the inspection of FRP-RC elements ^1^.

Slab	Parameters		Selected NDTs	
			GPR	PAU
O	Damaged GFRP bars	Rupture	LD	ND
		Cross-sectional property loss	LD	ND
		Debonding	LD	ND
P	Damaged CFRP strands	Rupture	LD	LD
		Cross-sectional property loss	LD	LD
		Debonding	LD	LD
	Damaged BFRP bar	Rupture	ND	ND
		Cross-sectional property loss	ND	ND
		Debonding	ND	ND
	Damaged steel bar	Rupture	D	D
		Cross-sectional property loss	D	D
		Debonding	D	D
Q	Defects/damage in concrete	Horizontal delamination	D	D
		Vertical Cracks	LD	LD
		Voids	LD	LD

Note: D = detectable; LD = limited detectability (based on depth); ND = not detectable. ^1^ The results were obtained from slab specimens with a maximum thickness of 7 inches and a maximum reinforcement depth of up to 4.5 inches.

## Data Availability

The data presented in this study are available by request from the corresponding author.
